# The Diversity and Similarity of Transmembrane Trimerization of TNF Receptors

**DOI:** 10.3389/fcell.2020.569684

**Published:** 2020-10-14

**Authors:** Linlin Zhao, Qingshan Fu, Liqiang Pan, Alessandro Piai, James J. Chou

**Affiliations:** Department of Biological Chemistry and Molecular Pharmacology, Harvard Medical School, Boston, MA, United States

**Keywords:** TNFR1, transmembrane domain, oligomerization, receptor activation, NMR

## Abstract

Receptors in the tumor necrosis factor receptor superfamily (TNFRSF) regulate proliferation of immune cells or induce programmed cell death, and many of them are candidates for antibody-based immunotherapy. Previous studies on several death receptors in the TNFRSF including Fas, p75NTR, and DR5 showed that the transmembrane helix (TMH) of these receptors can specifically oligomerize and their oligomeric states have direct consequences on receptor activation, suggesting a much more active role of TMH in receptor signaling than previously appreciated. Here, we report the structure of the TMH of TNFR1, another well studied member of the TNFRSF, in neutral bicelles that mimic a lipid bilayer. We find that TNFR1 TMH forms a defined trimeric complex in bicelles, and no evidences of higher-order clustering of trimers have been detected. Unexpectedly, a conserved proline, which is critical for Fas TMH trimerization, does not appear to play an important role in TNFR1 TMH trimerization, which is instead mediated by a glycine near the middle of the TMH. Further, TNFR1 TMH trimer shows a larger hydrophobic core than that of Fas or DR5, with four layers of hydrophobic interaction along the threefold axis. Comparison of the TNFR1 TMH structure with that of Fas and DR5 reveals reassuring similarities that have functional implications but also significant structural diversity that warrants systematic investigation of TMH oligomerization property for other members of the TNFRSF.

## Introduction

**FIGURE 1:**
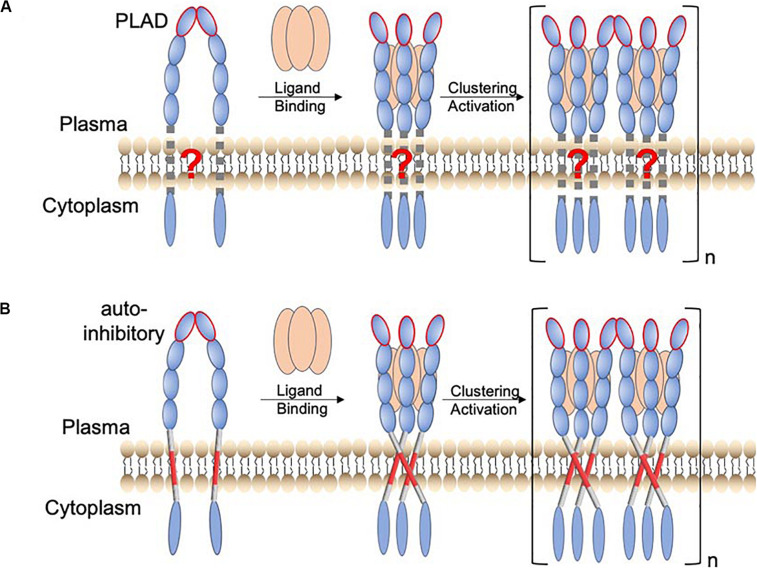
Proposed function of TMH oligomerization in receptor activation of the TNFRSF. **(A)** Schematic of a TNFR activation mechanism in which the TMH only plays the passive role of membrane anchoring. The resting state involves preligand receptor association mediated mainly by the homodimeric interaction of the pre-ligand association domain (PLAD; ellipsoid with red edge). Binding of the trimeric ligand causes receptor trimerization, which in turn leads to higher-order receptor clustering and activation. **(B)** A revised receptor activation mechanism in which the TMH specifically oligomerizes, positioning the intracellular domains to form signaling-compatible complexes. In this case, the preligand receptor association prevents the specific TMH oligomerization that drives downstream signaling. The consequence of ligand binding is to overcome this inhibitory constraint.

Receptors in the tumor necrosis factor receptor superfamily (TNFRSF) are Type I transmembrane proteins with an ectodomain (ECD) composed of multiple cysteine-rich domains (CRDs), a transmembrane helix (TMH), and an intracellular region that specifically interacts with signaling adaptors such as the Fas-associated death domain (FADD), the TNFR1-associated death domain (TRADD), or the TNFR-associated factors (TRAFs) (Baker and Reddy, 1998). In-depth understanding of the mechanism by which these receptors are activated is becoming increasingly important, as many of them are targets for antibody-based immunotherapy (Chaudhary et al., 1997; Sheridan et al., 1997; Hatzoglou et al., 2000; Rogers et al., 2001; Cooper et al., 2002; Ashkenazi, 2008; Croft et al., 2013). Early functional and structural studies on TNFR1 and Fas have suggested a general model of receptor activation in which the binding of the trimeric ligand causes the receptor ECD to trimerize, allowing subsequent clustering of the intracellular domains that recruits and activates downstream signaling proteins (Wajant, 2002; Vanamee and Faustman, 2018) (Figure 1A; schematic of the receptor activation model without considering the TMH). This mechanism, however, did not include the role of the TMH but disease mutations in the TMH of Fas have been documented (Gronbaek et al., 1998; Lee et al., 2000). We have thus undertaken structural and functional investigation of the TMHs of members of the TNFRSF.

Previous studies have already suggested the function of TMH dimerization in the signaling of death receptors p75NTR (Goh et al., 2018) and DR5 (Valley et al., 2012). We found that Fas TMH in bicelles (*q* = 0.5) forms a defined trimer around a proline-containing signature sequence, and disruptive mutations for TMH trimerization severely attenuate Fas ligand (FasL)-induced signaling (Fu et al., 2016), suggesting that specific trimerization of TMH is essential for positioning the intracellular DDs to cluster and form the signaling-compatible complex. More recently, we made another unexpected finding that the TMH of DR5 not only trimerizes but also dimerizes via a GXXXG motif (MacKenzie et al., 1997; Trenker et al., 2015), resulting in the formation of dimer–trimer interaction network (Pan et al., 2019). This higher-order clustering of TMH is also critical for DR5 activation as single mutations that disrupt either trimerization or dimerization abolish ligand-induced receptor activation (Pan et al., 2019). More strikingly, proteolytic removal of the ECD of DR5, which deletes the extracellular constraints on the TMH, can activate DR5 to the same extent as its native ligand (TRAIL) (Pan et al., 2019). This result, combined with TMH clustering, suggests that the ECD adopts a preligand conformation that precludes the TMH oligomerization essential for downstream signaling and that the primary consequence of ligand binding is to overcome this inhibitory constraint (Figure 1B; schematic of receptor activation including the role of the TMH).

The mechanism in Figure 1B could have major therapeutic implication, as it suggests that a true agonistic antibody must be able to break the autoinhibitory, preligand association of receptor ECD so that the TMH can freely oligomerize, positioning the intracellular region for efficient formation of signaling capable clusters. Consistent with this mechanism, proteolytic removal of ECD can directly activate DR5 because DR5 TMH alone can form cluster of trimers via the GXXXG dimerization motif. TNFR2 and OX40 can also be activated by proteolytic removal of ECD (Pan et al., 2019), and interestingly, their TMHs also contain GXXXG. Conversely, if the TMH can form multimer of trimers, then disrupting the preligand ECD association by either soluble ligand or antibody should be sufficient to activate the receptor. Thus, a broader survey of the clustering properties of TMHs in the TNFRSF would evaluate the generality of the mechanism in Figure 1B while potentially discovering exceptions to the rule.

In this study, we examined the TMH of TNFR1 in bicelles that mimic a lipid bilayer. We used biochemical method to show that TNFR1 TMH forms homogeneous trimers in neutral lipid bicelles. We then used NMR to determine the structure of the TMH trimer. The TMH trimerization of TNFR1 shows features that are strongly distinct from that of Fas and DR5, implying the general unpredictability of TMH trimerization for receptors in the TNFRSF.

## Results

### Amino Acid Sequences of TNFR1 TMH

**FIGURE 2:**
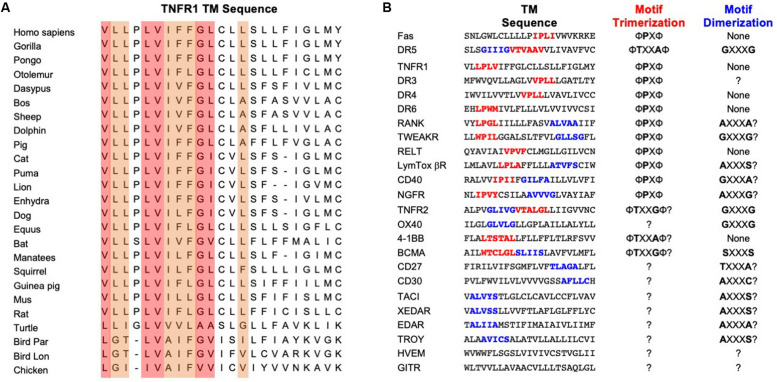
Amino acid conservation of TNFR1 TMH and TMH sequences of other members of the TNFRSF. **(A)** Alignment of TNFR1 TMH sequences from various organisms generated using the ClustalX2 program (Larkin et al., 2007). The most conserved positions are shaded in light red; the secondary higher identity positions are shaded in light orange. **(B)** Comparison of TMH sequences from the TNFRSF. Proposed trimerization and dimerization motifs are shown in red and blue, respectively. The “?” indicates unknown or highly speculative.

Sequence alignment of TNFR1 TMH from different organisms shows a few interesting and useful features (Figure 2A). The N-terminal half (residues 212-222) is much more conserved than the C-terminal half (residues 223-234). Previous structural analysis of the TMHs of Fas and DR5 revealed proline and threonine/alanine-based motifs, respectively, that mediate TMH trimerization, and these motifs indeed can be found in many of the TNFRSF members, including TNFR1 (Figure 2B). The Fas TMH structure shows a proline-containing signature sequence (ΦPxΦ) that drives TMH trimerization, where Φ represents hydrophobic residues, P is proline, and x can be any apolar residues except for proline and glycine. TNFR1 TMH also contains a LP^215^LV that fits the ΦPxΦ description but is suspiciously close to the N-terminal end of the TMH. Hence, it is important to examine whether the proline plays a role in TNFR1 TMH oligomerization.

### Protein Construct for Structural Analysis

The human TNFR1 TM fragment, residue 209–238, designated TNFR1 TMH, was selected for structural study. The residue C223 in the middle of the TM region was mutated to alanine to avoid artificial disulfide bond formation in solution during protein reconstitution. In addition, M233 is incompatible with the TrpLE expression system, which requires cleavage at the N-terminal methionine to separate the TrpLE and the TM fragment; it is also poorly conserved (Supplementary Figure S1A). Therefore, M233 was mutated to alanine as well. The C223, however, is quite conserved as shown in Supplementary Figure S1A, suggesting that it could participate in oligomerization. Hence, this was initially a risky mutation for facilitating sample preparation but, in retrospect, turned out to be harmless as residue 223 is lipid-facing (Supplementary Figure S1B) and on the opposite side of the helix–helix packing interface (described later in the article after structure determination).

### Structure Determination in Bicelles That Mimic a Lipid Bilayer

**FIGURE 3:**
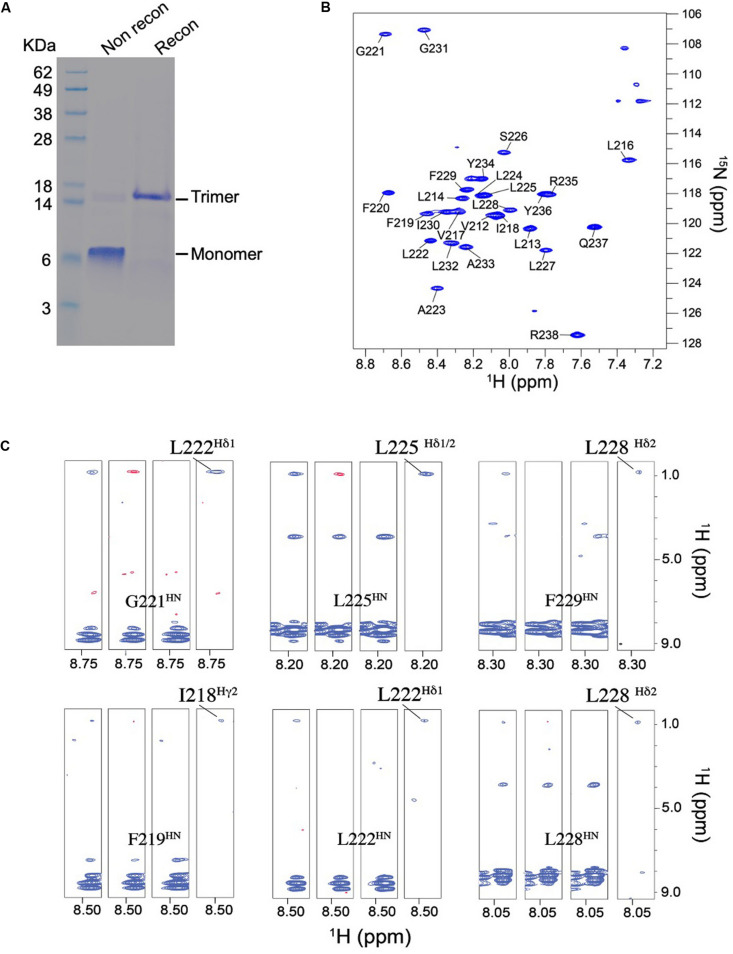
Biochemical and NMR characterizations of the TNFR1 TMH. **(A)** Oligomerization of TNFR1 TMH in bicelles analyzed by standard SDS-PAGE. The gel lanes from left to right are: (1) MW markers; (2) purified TNFR1 TMH powder without reconstitution; (3) TNFR1 TMH reconstituted in DMPC- DH_6_PC bicelles (*q* = 0.5). Both TNFR1 TMH samples were dissolved in gel loading buffer prior to SDS-PAGE. **(B)** The ^1^H-^15^N TROSY-HSQC spectrum of (^15^N,^13^C, ^2^H)-labeled TNFR TMH reconstituted in same bicelles, recorded at ^1^H frequency of 600 MHz at 303 K. **(C)** Detection of inter-chain NOEs. Residue-specific strips from the *J*_*CH*_-modulated NOESY (NOE mixing time = 200 ms) recorded at 800 MHz and 303 K. The sample comprises 50% (^15^N,^2^H)-labeled and 50% (^1^H,^13^C)-labeled TNFR1 TMH. For each selected residue, four strips are shown from left to right: (1) positive inter-NOEs, blue; (2) negative inter-NOEs, red; (3) inter-NOEs are canceled [(1) + (2)]; (4) inter-NOEs are selected [(1) - (2)].

TNFR1 TMH was expressed, purified, and reconstituted in neutral lipid bicelles as previously described (Fu et al., 2019). The purified protein fragment was reconstituted in DMPC-DH_6_PC bicelles with *q* = 0.5, where *q* is the molar ratio of DMPC/DH_6_PC. The final NMR sample contains ∼0.7 mM TNFR1, 50 mM DMPC, 100 mM DH_6_PC, and 20 mM phosphate buffer (pH 6.8). At *q* = 0.5, the diameter of the planar bilayer region of the bicelles is ∼45 Å (Sanders and Schwonek, 1992; Glover et al., 2001). As in the case of Fas TMH, the bicelle-reconstituted TNFR1 TMH ran on SDS-PAGE as trimers (theoretical MW of TNFR1 TMH is ∼3.4 kDa; trimer is between 14 and 18 KDa), whereas unreconstituted peptide migrated as monomers on the gel (Figure 3A), providing the direct evidence that TNFR1 TMH spontaneously formed homotrimers in bicelles and that the trimeric complexes, once formed, can resist the strong denaturing environment of SDS-PAGE. The reconstituted TNFR1 TMH in bicelles generated TROSY-HSQC spectrum with good chemical shift dispersion and peak homogeneity (Figure 3B and Supplementary Figure S2) and in combination with the SDS-PAGE result indicates that TNFR1 TMH in bicelles is a homogeneous trimer suitable for full-scale structure determination.

The NMR structure of the TNFR1 TMH trimer was determined using a published protocol (Fu et al., 2019). Briefly, the protocol involves (1) construction of a preliminary monomer structure with local nuclear Overhauser effect (NOE) restraints and backbone dihedral angles derived from chemical shift values (using TALOS+ Shen et al., 2009), (2) obtaining a unique structural solution of the trimer with inter-chain NOE restraints derived from mixed isotopically labeled sample, and (3) refinement of the trimer structure by further assignment of self-consistent NOE restraints. Assignment of the H^*N*^, N, C’, and C^α^ resonances was achieved for residues 212–238 except for that of P215. For initially identifying inter-chain contacts, we used mixed samples in which half of the monomers are (^15^N, ^2^H)-labeled and the other half ^13^C-labeled, and performed the *J*_*CH*_-modulated NOE experiment (Fu et al., 2016, 2018) to detect exclusively NOEs between the ^15^N-attached protons of one subunit and ^13^C-attached protons of the neighboring subunits. This type of inter-chain NOE peaks is positive in *J*_*CH*_-unmodulated spectrum and negative in the *J*_*CH*_-modulated spectrum (see examples in Figure 3C). The 15 lowest energy structures of 100 calculated converged to root-mean-square deviation (RMSD) of ∼0.862 and ∼1.411 Å for backbone and all heavy atoms, respectively (Supplementary Figure S3 and Supplementary Table S1).

### Structure of the TMH Trimer of Human TNFR1

**FIGURE 4:**
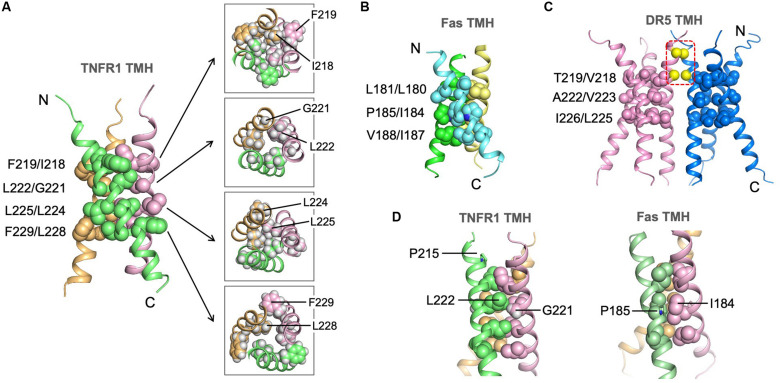
Structure of the TNFR1 TMH trimer in bicelles and comparison with TMHs of Fas and DR5. **(A)** Ribbon representation (left) of the trimeric TNFR1 TMH with core residues highlighted (side-chain heavy atoms shown as spheres). The sidechain packing at four different levels along the threefold axis is further illustrated with sectional top views of the trimer (right) (sidechain heavy atoms and protons included). **(B)** The structure of the Fas TMH trimer with core residues at three layers along the TM helices. The sidechain heavy atoms are shown as spheres. **(C)** The dimer-of-trimer structure of DR5 TMH. The trimer packing is displayed as in **(A)** and **(B)**. In addition, the Cα atoms of G213 and G217 in the dimer interface are shown as yellow spheres. **(D)** Comparison of the glycine-mediated helical packing of TNFR1 TMH (left) and the proline-mediated helical packing of Fas TMH (right). Residues in the hydrophobic core are highlighted (side-chain heavy atoms shown as spheres).

The trimeric structure of TNFR1 TMH shows an extensive hydrophobic core formed by bulky hydrophobic amino acids such as leucine and isoleucine. In this regard, it is similar to the Fas TMH structure. TNFR1 TMH trimer, however, shows a more extended hydrophobic core as there appears to be four layers of hydrophobic interaction along the 3-fold axis, including interactions between F219 and I218, between L222 and G221, between L225 and L224, and between F229 and L228 (Figure 4A). The core interactions involving I218 and L228 are likely weaker than those of central residues (e.g., G221, L225) because their associated inter-chain NOEs are much weaker (see Figure 3C). The hydrophobic core of the Fas TMH trimer comprises three layers of hydrophobic interaction: L181-L180, P185-I184, and V188-I187 (Figure 4B). It is also interesting to mention that the hydrophobic core the DR5 TMH trimer is formed mostly with small amino acids such as alanine and threonine (Figure 4C). Another major difference of DR5 TMH is the presence of the GXXXG motif (MacKenzie et al., 1997; Trenker et al., 2015) that allows DR5 TMH to form multimer of trimers.

Although the LP^215^LV fits the ΦPxΦ motif that mediates Fas TMH trimerization, we did not detect any significant inter-chain NOEs around P215, and this is consistent with the fact that P215 is not involved in helix-helix packing in our structure. Instead, the structure suggests that G221 near the middle of the TMH plays the important role of allowing close van der Waals (VDW) contact with L222 of the neighboring chain, which appears to allow close packing of I218 and L225 above and below it, respectively, from the three chains (Figure 4D). In this regard, G221 seems to serve the role of P185 in the Fas TMH trimer in allowing VDW contact with I184 of the neighboring chain (Figure 4D).

### Residues Important for TNFR1 TMH Trimerization

To examine the structure independently by mutagenesis, we generated three single mutations—P215Y, G221Y, and L225Y—and evaluated their effect on TMH trimerization (Supplementary Figure S4). Mutating P215 to tyrosine has essentially no effect on TMH trimerization in bicelles, further supporting the structural conclusion in Figure 4D that this relatively conserved proline does not play a role in helix–helix packing. As shown in Figure 4A, G221 is involved in close inter-helical packing with L222 and mutating G221 to the bulky tyrosine is expected to disrupt such packing. Indeed, the G221Y mutant showed a dominant dimer band and a very minor trimer band in SDS-PAGE, suggesting that this mutant cannot form specific trimers but could aggregate as non-specific dimers. Finally, the mutation L225Y almost completely abolished trimerization and migrated as monomers. This is consistent with L225 forming the most compact hydrophobic core along the TMH (Figure 4A). Overall, the oligomeric properties of the three mutants agree well with the NMR structure.

## Discussion

We have shown that the TMH of TNFR1 forms intimately assembled trimeric complex in a lipid bilayer environment. We initially thought that the LP^215^LV sequence near the N-terminal end fits the description of the ΦPxΦ motif that mediates Fas TMH trimerization and thus could be the key element of TMH trimerization. But our structure and mutagenesis data indicate otherwise. Instead, G221 near the middle of the TMH appears to be important as it allows intimate contact with the adjacent chain at this position. In this context, the structural role of the glycine is similar to the proline of the ΦPxΦ motif, which is to permit VDW contact with the neighboring chain such that the hydrophobic core of the trimer can form. We also emphasize that although the hydrophobic packing along the threefold axis of the TNFR1 TMH trimer appears to be quite extensive (comprising four layers of interactions), only the central interactions L222-G221 and L225-L224 show very intense inter-chain NOEs, suggesting the trimerization at the levels of I218 and L228 are weak and possibly more dynamic. In particular, L228 and generally the C-terminal region of the TMH after L225 are poorly conserved.

Like Fas, TNFR1 TMH can only form trimer but not higher order cluster of trimers as the dimeric interaction is lacking. But unlike Fas, TNFR1 can be activated by soluble TNF ligand, whereas Fas can only be efficiently activated by crosslinked Fas ligand (FasL) (Banner et al., 1993; Wajant et al., 2003); when the membrane-bound FasL is shedded to become soluble, it can no longer activate Fas (Schneider et al., 1998; Tanaka et al., 1998). In the context of ligand requirement, TNFR1 is more similar to DR5, which can be efficiently activated by soluble ligand (TRAIL). We have previously shown that DR5 can be activated by soluble TRAIL owing to its TMH’s capacity to form higher-order dimer-trimer network to drive receptor clustering when unconstrained by the autoinhibitory, preligand association of the ECD (Pan et al., 2019). TNFR1 TMH, however, does not have the capacity to form cluster of trimers. We thus speculate that the previously suggested dimeric interactions of TNFR1 ECD in crystal structures (Naismith et al., 1996) could complement TMH trimerization by allowing clustering of trimeric receptors. It has been shown that the first CRD of TNFR1 (CDR1) is responsible for mediating receptor association on the cell surface in the absence of ligand and is thus known as the preligand association domain (PLAD) (Chan, 2000; Karathanasis et al., 2020; Weinelt et al., 2020). Further, the crystal structure of receptor–ligand complex (Banner et al., 1993) shows that the CDR1 of TNFR1 is not involved in ligand binding, although its presence appears to be important for the optimal binding of the ligand by CRD2 and CRD3 (Branschadel et al., 2010). These evidences suggest that the CRD1 of TNFR1 can provide the dimeric interaction for achieving higher-order receptor dimer–trimer network. Indeed, soluble TNFR1 CRD1 has been used to compete with CRD1-mediated receptor association, which inhibits receptor clustering and activation, as a new anti-arthritis treatment strategy (Deng, 2007). In addition to the ECD, the self-interaction of the intracellular domains could also contribute to receptor clustering and this type of interaction has been well characterized, for example, for death receptors such as DR3 and Fas (Scott et al., 2009; Wang et al., 2010; Yin et al., 2019).

Finally, the premise of the above analysis is that the trimerization of TNFR1 TMH is required for ligand-induced signaling. Unfortunately, to the best of our knowledge, there have been no report of naturally occurring, disease-causing mutations in the TMH of TNFR1 that would indicate the function of TMH oligomerization in receptor activation. It is thus important to perform functional mutagenesis of the TMH in the context of the full-length TNFR1. The TMH structure reported in this article should guide this effort.

## Conclusion

We have thus far determined the TMH structures for Fas, DR5, and TNFR1 in essentially lipid bilayer environment. While they show obvious similarities, there are significant differences that make sequence-based structural prediction extremely difficult. One fundamental property shared by the three receptors is the ability of the TMH to spontaneously form defined trimer in lipid bilayer, although the TMH of DR5, in addition, can dimerize via the GXXXG signature sequence. Another similarity is that these trimers are all stabilized by hydrophobic interactions in the core of the assembly, and the intimate helical packing is made possible by small amino acids such as proline, glycine, alanine, or threonine. But, the nature of the hydrophobic core formation is where the biggest differences reside among these TMH structures. While the larger hydrophobic amino acids such as leucine, isoleucine, and valine make up the cores of Fas and TNFR1 TMH trimers, the small alanine and threonine appear to dominate the hydrophobic core of DR5 TMH trimer. In the case of Fas TMH, the critical proline not only facilitates close helix–helix packing but also introduces backbone malleability for accommodating the hydrophobic core (Fu et al., 2016). Although TNFR1 TMH also has a relatively conserved proline, it is the glycine that permits intimate helical packing. The GXXXG or small-XXX-small motif has been rather consistent in predicting TMH dimerization. Determinants for TMH trimerization, however, could be highly diverse. Hence, it remains important to experimentally survey the oligomerization properties of TMHs of other members of the TNFRSF to gain a broad understanding of the functional roles of TMH in receptor activation.

## Materials and Methods

### Protein Expression and Purification

The DNA corresponding to the human TNFR1 (isoform 1) fragment, residues 209–238, designated TNFR1 TMH, was synthesized by GenScript (Piscataway, NJ, United States). Residues C223 and M233 were mutated to alanines to facilitate expression and purification. The protein expression construct was created by fusing the TNFR1 TMH fragment to the C terminus of the His9-TrpLE expression sequence in the pMM-LR6 vector, with an added methionine in-between for cleavage by cyanogen bromide. For NMR sample preparation, transformed *Escherichia coli* strain BL21 (DE3) bacteria were grown in M9 minimal media supplemented with centrum multivitamins and stable isotopes. Cultures were grown at 37°C to an absorbance of ∼0.6 at 600 nm and cooled to 25°C before induction with 500 μM isopropyl β-D-thiogalactopyranoside at 25°C for overnight. For fully deuterated proteins, bacterial cultures were grown in 99.8% D_2_O (Sigma Aldrich, St. Louis, MO, United States) with deuterated glucose (Cambridge Isotope Laboratories, Tewksbury, MA, United States). The TNFR1 TMH protein was extracted, cleaved by cyanogen bromide, purified and lyophilized as described (Fu et al., 2016). Bacteria were harvested and resuspended in 50 mM Tris-HCl (pH 8.0) and 200 mM NaCl. The bacteria were sonicated twice and centrifuged at 40,000×g for 30 min to collect inclusion body pellets. The inclusion body pellets were dissolved in 6 M guanidine HCl, 50 mM Tris (pH 8.0), 100 mM NaCl, and 1% (v/v) Triton X-100. The solubilized solution of inclusion body was loaded to a Ni^2+^ affinity column (Sigma), washed with 8 M urea solution and distilled water, and eluted with 70% (v/v) formic acid. The fusion protein was cleaved at the methionine position by cyanogen bromide (0.1 g/mL) to release the TNFR1 TMH peptide. The cleaved peptide was then precipitated in water, lyophilized, dissolved in 50% formic acid, and loaded to a Zorbax SB-C3 column (Agilent), equilibrated in Buffer A [5% isopropanol, 0.1% trifluoroacetic acid (TFA)]. TNFR1 TMH was separated from the unwanted species in a gradient of 50–100% Buffer B (25% acetonitrile, 75% isopropanol, 0.1% TFA). The eluted TNFR1 TMH was lyophilized for storage.

### NMR Sample Preparation in Bicelle

To reconstitute TNFR1 TMH in bicelles, 1∼2 mg of the purified and lyophilized protein was mixed with 9 mg 1,2-Dimyristoyl-sn-Glycero-3-Phosphocholine (DMPC, protonated or deuterated from Avanti Polar Lipids, Alabaster, AL, United States) and dissolved in 1,1,1,3,3,3-hexafluoro-2-propanol. The mixture was slowly dried to a thin film under nitrogen stream, followed by overnight lyophilization. The dried thin film was redissolved in 2 mL of 8 M urea containing ∼27 mg 1,2-Dihexanoyl-sn-Glycero-3-Phosphocholine (DH_6_PC, protonated or deuterated from Avanti Polar Lipids). The mixture was dialyzed twice against a 20 mM phosphate buffer (pH 6.8) (1 L each time) to remove the denaturant, and 10 mg DH_6_PC was added to the sample before the second dialysis to compensate its loss. The DMPC:DH_6_PC ratio was monitored by 1D NMR throughout the reconstitution process. If needed, additional DH_6_PC was added to make the final DMPC:DH_6_PC ratio between 0.5 and 0.6. The sample was concentrated using Centricon (EMD Millipore, Billerica, MA, United States) to ∼350 μL. The final NMR sample contained ∼0.7 mM TNFR1 TMH (monomer), ∼50 mM DMPC, ∼100 mM DH_6_PC, 20 mM phosphate buffer (pH 6.8), 0.02% NaN_3_ and 5% D_2_O. For all NOE experiments, the protein was reconstituted using DMPC and DH_6_PC with deuterated acyl chains (Avanti Polar Lipids).

### SDS-PAGE Analysis of TMH Oligomerization

For SDS-PAGE analysis of the bicelle-reconstituted samples, lyophilized protein (2 mg) was dissolved in hexafluoro-isopropanol (HFIP) with 2 mg DMPC, followed by drying of the solution under a nitrogen stream to achieve a thin film. The thin film was then dissolved in 1 ml of an 8 M urea solution containing approximately 6 mg DH_6_PC, followed by dialysis against 20 mM sodium phosphate buffer (pH 6.8) to remove the denaturant. After dialysis, DH_6_PC was added to adjust the ratio of DMPC:DH_6_PC to approximately 1:2. To perform gel electrophoresis, 20 μL of the reconstitution sample was mixed with 5 μL of 4× (dilution) LDS loading buffer (Invitrogen, Catalog No.: NP0007) without heating or other reducing agents, and loaded to an Invitrogen NuPAGE 12% gel (Catalog No.: NP0342BOX). The gel was run at 200 V on ice for 30 min. For SDS-PAGE analysis of the unreconstituted samples, lyophilized protein powder suspended in 1× LDS loading buffer (Invitrogen, Catalog No.: NP0007) was heated at 100°C for 10 min and loaded to an Invitrogen NuPAGE 12% gel (Catalog No.: NP0342BOX).

### NMR Resonance Assignment

All NMR data was recorded at 30°C (303 K) on Bruker spectrometers operating at ^1^H frequency of 800 MHz, 750 MHz, or 600 MHz and equipped with cryogenic probes. NMR data were processed using NMRPipe (Delaglio et al., 1995), and spectra are analyzed using XEASY (Bartels et al., 1995) and CcpNmr (Vranken et al., 2005). Triple resonance experiments were collected at ^1^H frequency of 600 MHz using a (^15^N, ^13^C, ∼85% ^2^H)-labeled sample. Sequence-specific assignment of backbone H^*N*^, ^15^N, ^13^C^α^, and ^13^C’ resonances was accomplished using 3D TROSY-based HNCA, HN(CO)CA, HN(CA)CO and HNCO experiments (Salzmann et al., 1999). The aliphatic and aromatic resonances of the protein side chains were assigned using the 3D ^15^N-edited NOESY-TROSY-HSQC (τ_*NOE*_ = 100 ms) and 3D ^13^C-edited NOESY-HSQC (τ_*NOE*_ = 150 ms) spectra, recorded at ^1^H frequency of 750 MHz using a (^15^N, ^13^C)-labeled protein sample in deuterated bicelles. For assigning inter-chain distance restraints, the *J*_*CH*_-modulated NOE experiment (Fu et al., 2019) was performed to exclusively detect inter-chain NOEs between the ^15^N-attached protons of one chain and the ^13^C-attached protons of the neighboring chains, using a mixed sample containing 50% (^15^N, ^2^H)-labeled and 50% ^13^C-labeled protein. In this experiment, two interleaved spectra were recorded with different times of *J*_*CH*_ evolution (*J*_*CH*_ = 0 ms and *J*_*CH*_ = 8 ms) before the NOE mixing. Subtraction of the two spectra allowed selection of the inter-chain NOE crosspeaks.

### Structure Calculation

The structures were generated using the program XPLOR-NIH (Schwieters et al., 2003). First, the monomer structure was generated using the short-range NOE restraints and the backbone dihedral restraints derived from the backbone ^15^N, ^1^H, ^13^Cα, and ^13^C’ chemical shifts [using the TALOS + program (Shen et al., 2009)]. The ^13^C^α^ secondary chemical shifts of TNFR1 TMH are shown in Supplementary Figure S2B, providing a secondary structure mapping of the TM fragment. Second, the monomer structure and inter-chain NOE restraints were used with the ExSSO program (Yang et al., 2017) to generate a unique solution of trimeric assembly. Finally, the initial trimer solution was fed to the XPLOR-NIH for iterative refinement against all NMR restraints, including the newly assigned self-consistent inter-chain NOEs from each iteration.

For each inter-chain restraint between two adjacent chains, three identical distance restraints were assigned respectively to all pairs of neighboring chains to satisfy the condition of C3 rotational symmetry. The XPLOR refinement used a simulated annealing (SA) protocol in which the temperature in the bath was cooled from 1000 to 200 K with steps of 20 K. The NOE restraints were enforced by flat-well harmonic potentials, with the force constant ramped from 2 to 30 kcal/mol Å^–2^ during annealing. Backbone dihedral angle restraints were taken from the “GOOD” dihedral angles from TALOS+, all with a flat-well (± the corresponding uncertainties from TALOS+) harmonic potential with force constant ramped from 5 to 1000 kcal/mol rad^–2^. A total of 100 structures were calculated and 15 lowest energy structures were selected as the final structural ensemble (Supplementary Figure S3 and Supplementary Table S1).

## Data Availability Statement

The datasets presented in this study can be found in online repositories. The atomic structure coordinate and structural constraints have been deposited in the Protein Data Bank (PDB), accession number 7K7A. The chemical shift values have been deposited in the Biological Magnetic Resonance Data Bank (BMRB), accession number 30799.

## Author Contributions

LZ and JC conceived the study. LZ, QF, and LP prepared samples for NMR and biochemical studies. LZ, AP, and JC collected and analyzed the NMR data and/or determined the structures. JC and LZ wrote the manuscript. All authors contributed to the editing of the manuscript.

## Conflict of Interest

The authors declare that the research was conducted in the absence of any commercial or financial relationships that could be construed as a potential conflict of interest.
