# Supplementary material for: The Diversity and Similarity of Transmembrane Trimerization of TNF Receptors
Source: Front Cell Dev Biol. 2020 Oct 14;8:569684. doi: 10.3389/fcell.2020.569684 (PMC7591462; doi:10.3389/fcell.2020.569684)
Supplement: Supplementary file 1 [file Data_Sheet_1.PDF]

Supporting Information

The diversity and similarity of transmembrane trimerization of TNF receptors

Linlin Zhao, Qingshan Fu, Liqiang Pan, Alessandro Piai, and James J. Chou

Table S1. NMR and refinement statistics

**NMR distance and dihedral constraints** [a]	TMH (209-238)
Distance constraints from NOE	540
Short-range intramolecular ($\|i-j\| \leq 4$)	168 x 3
Long-range intramolecular ($\|i-j\| \geq 5$)	0
Intermolecular	12 x 3
Total dihedral angle restraints [b]	150
ϕ (TALOS)	25 x 3
ψ (TALOS)	25 x 3
**Structure statistics** [c]	
Violations (mean ± s.d.)	
Distance constraints (Å)	0.070 ± 0.006
Dihedral angle constraints (°)	0.230 ± 0.039
Deviations from idealized geometry	
Bond lengths (Å)	0.005 ± 0.000
Bond angles (°)	0.615 ± 0.011
Impropers (°)	0.335 ± 0.019
Average pairwise r.m.s. deviation (Å) [d]	
Heavy	1.411
Backbone	0.862

[a] The numbers of constraints are summed over all three subunits.

[b] Backbone ϕ and ψ restraints and their respective uncertainties were obtained from the “GOOD” dihedrals generated by the TALOS+ program (42) based on the backbone chemical shift values.

[c] Statistics are calculated and averaged over an ensemble of the 15 lowest energy structures out of 100 calculated structures.

[d] The precision of the atomic coordinates is defined as the average r.m.s. difference between the 15 final structures and their mean coordinates.

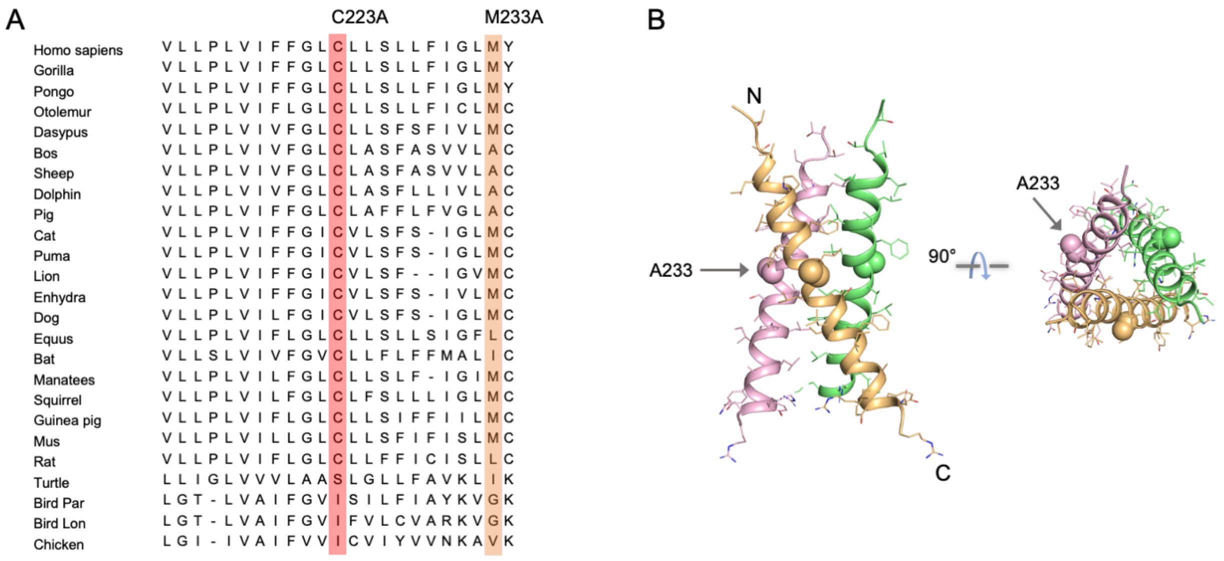

Figure S1. Conservation of Cys223 and Met233 and suitability for mutagenesis for enabling structural studies. (**A**) Alignment of TNFR1 TMH sequences from various organisms generated using the ClustalX2 program (44), showing relative conservation of Cys223 and Met233. (**B**) The trimeric structure of the TNFR1 TMH, described later in the paper, shows that residue 223 is not involved in TMH trimerization.

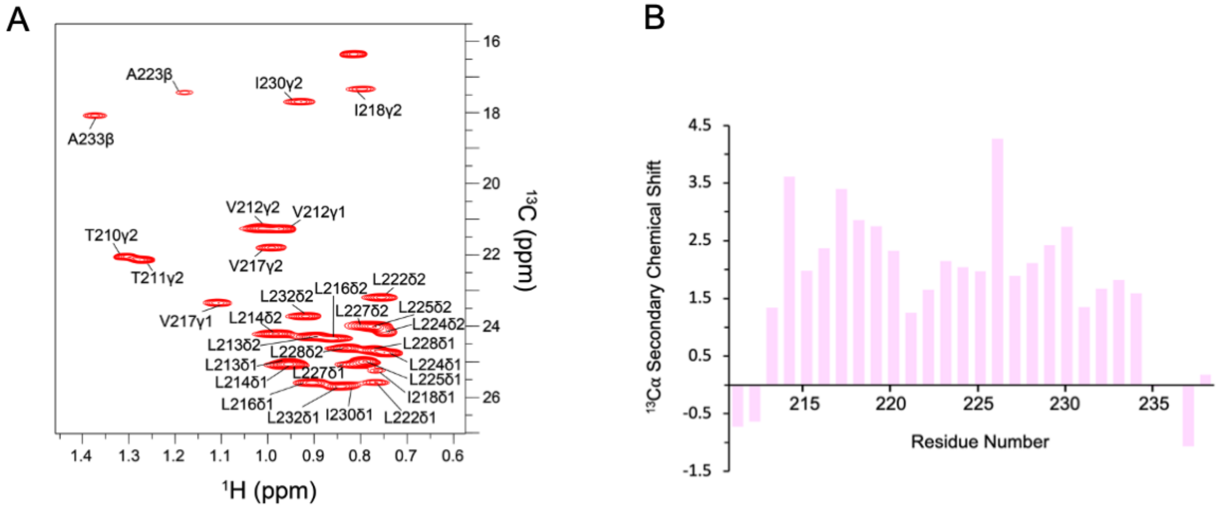

Figure S2. Methyl resonances and secondary chemical shifts of TNFR1 TMH in bicelles. (**A**) The methyl group region of the 2D ^{1}H-^{13}C HSQC with 28 ms constant-time ^{13}C evolution, recorded at ^{1}H frequency of 750 MHz, using (^{15}N, ^{13}C)-labeled protein. (**B**) ^{13}Cα secondary chemical shift analysis of TNFR1 TMH in bicelles. The values are generated using the TALOS+ program (42).

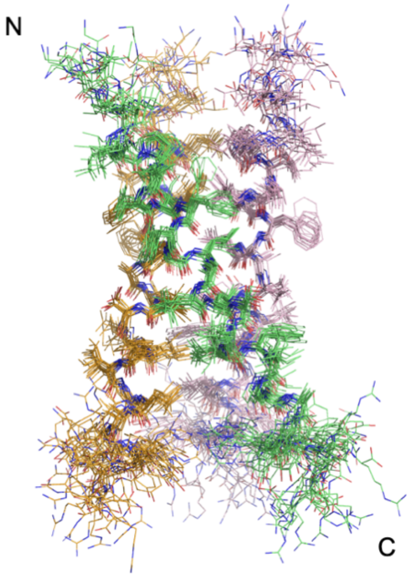

Figure S3. Structural convergence of TNFR1 TMH in bicelles. Ensemble of 15 lowest energy structures from 100 structures calculated using NMR-derived restraints. Protons are not displayed.

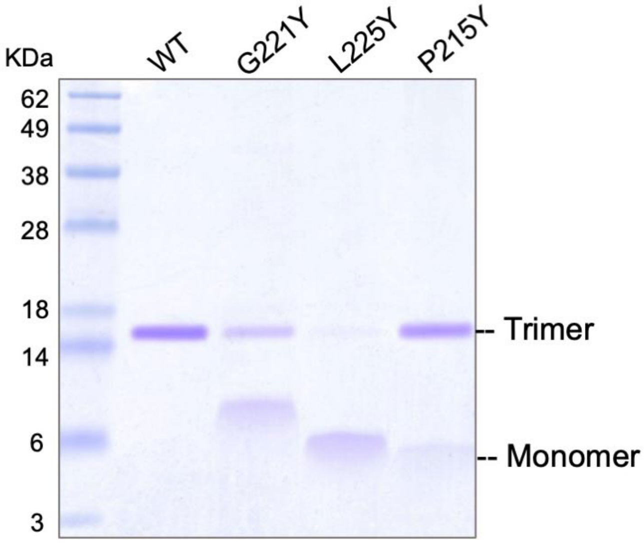

Figure S4. Effects of single mutations on trimerization of the TNFR1 TMH. SDS-PAGE of bicelle-reconstituted human TNFR1 TMH and its mutants showing the effect of single mutations on trimerization. Samples were run under non-denaturing conditions (see Materials and Methods for details of the gel electrophoresis).
